# Chameleon Effect of Universal Shade Composite Polymers in Repairing CAD/CAM Lithium Disilicate

**DOI:** 10.3390/ma18133020

**Published:** 2025-06-25

**Authors:** Gaetano Paolone, Giacomo Collivasone, Niccolò De Masi, Alicia Heinichen, Katia Greco, Enrico Gherlone, Giuseppe Cantatore

**Affiliations:** Dental School, IRCCS San Raffaele Hospital, Vita-Salute University, 20132 Milan, Italy; g.collivasone@gmail.com (G.C.); niccolo_99@icloud.com (N.D.M.); a.heinichen@studenti.unisr.it (A.H.); katiagreco@libero.it (K.G.); gherlone.enrico@hsr.it (E.G.); cantatore.giuseppe@hsr.it (G.C.)

**Keywords:** chameleon effect, universal shades, CAD/CAM, lithium disilicate, composite, repair, color adjustment potential

## Abstract

The aim was to assess the blending effect of universal-shade resin-based composites (RBCs) (Omnichroma—OC; Clearfil Majesty Universal–CM; Venus Pearl—V; Transcend—T) used for repair for lithium disilicate blocks. Fifteen parallelepiped-shaped (10.5 × 10.5 × 3 mm) specimens with four cavities (3 mm in diameter and 2 mm in depth) were designed from lithium disilicate CAD/CAM blocks (CEREC Tessera HT A3) and milled. Specimens were then randomly divided into five groups based on the five resin composite materials for cavity restoration (*n* = 12): Group 1, control group (F); Group 2 (T); Group 3 (OC); Group 4 (V); and Group 5 (CM). After surface conditioning, composite resins were applied to the ceramic surface. Color measurements were taken with a colorimeter in the center of the resin restoration and on the CAD/CAM block. Tristimulus values were converted to CIELab color coordinates and color differences were expressed in ΔE_00_ units using the CIEDE-2000 formula. F showed significantly better color matching (ΔE_00_ = 2.51 ± 0.64) in comparison to single-shade RBCs except T (ΔE_00_ = 2.55 ± 0.64). All groups exceeded perceptibility and acceptability thresholds. The control group presented higher color matching than the single shade universal composites except for Transcend.

## 1. Introduction

The introduction of the acid-etch technique in 1955 by Michael Buonocore [1], the bisphenol A-glycidyl methacrylate (Bis-GMA) by Dr. Bowen [2] in the early 1960s, and subsequently, the launching of the first commercial resin-based composite (RBC) set a milestone for adhesive dentistry. Ever since, constant modifications have been made to improve the properties of RBCs. Nowadays, clinicians have a plethora of composites with enhanced physical properties used to functionally and esthetically restore both anterior and posterior teeth. The tooth is composed of different tissues and the shade derives from the combination between them and light. Dentin is the dental tissue mainly responsible for its color [3], while enamel is a colorless and translucent tissue that acts as a filter [4], since the orientation of enamel rods causes light to be scattered. When a resin restoration is placed, a complex color interaction between dental tissues and RBCs takes place. According to several factors, RBCs may have a variable degree of camouflage with the dental substrate. Hall and Kafalias [5] reported this phenomenon as the “chameleon effect”. In the literature, other terms such as “color assimilation” [6], “blending effect” (BE) [7], or “color adjustment potential” (CAP) [8] are also used to describe the ability to blend with the surrounding tooth substrate. In the last decades, dental companies have launched different composite shades with similar optical properties to enamel and dentin, introducing composite layering techniques [9,10,11,12]. Clinicians need to stratify different restorative material to an adequate thickness and position to create an almost imperceptible restoration [13]. Koi et al. [14] reported that mastering the different layering concepts is time-consuming and a compelling task that depends on clinical expertise. Recently, to simplify clinical protocols, the industry has aimed to reduce the number of shades and universal shades have been introduced [14,15]. As reported by Altınışık and Özyurt [16], these new composites have the advantage of being able to simulate all shades of tooth color using only a single shade. Hence, manufacturers claim that these universal composite resins are able to have a true chameleon effect [17,18]. Perdigão et al. [19] reported that these new materials not only offer tremendous advantages but also revolutionize their promising use in clinical practice, particularly when compared to materials that require multiple shades to accomplish a successful restoration. Ceramics can also address the demands for cosmetic dentistry, as they are considered the material closest to dental structures. Thanks to their excellent mechanical, chemical, and optical properties [20,21], they can mimic the natural appearance of teeth. Above all, they offer high biocompatibility and stability [22]. To date, silicates, and in particular lithium disilicate, are one of the most widespread and versatile dental ceramics used [23]. However, two major drawbacks have to be considered, which are production costs and cyclic fatigue [24]. Common complications comprise chipping, cracking, and fracture [25,26,27], and represent an esthetical and functional problem for patients [28]. RBCs are the most widely used material for repair as they avoid the expense and time of remaking of ceramic [29]. The use of simplified restorative procedures, including universal composites, could facilitate clinicians’ procedures and increase the longevity of ceramic frameworks. The aim of this study was, therefore, to assess the chameleon effect of four universal shades used for repair of lithium disilicate prosthetic elements. The null hypothesis was that there is no difference in the ability to blend in for universal composites, compared to conventional RBCs.

## 2. Materials and Methods

Lithium disilicate CAD/CAM blocks (CEREC Tessera^®^ HT A3 C14, shade A3, Dentsply Sirona, Charlotte, CN, USA) were milled to create four cavities with a depth of 2 mm and a diameter of 3 mm. The specimens were then filled with the tested materials (*n* = 12). The characteristics and composition of the tested materials used in this study are presented in Table 1.

### 2.1. Specimen Preparation

**Figure 1:**
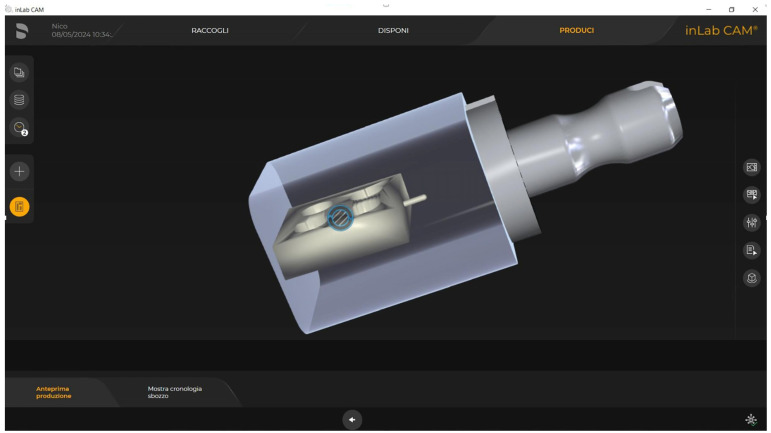
Preview of the specimens’ design.

**Figure 2:**
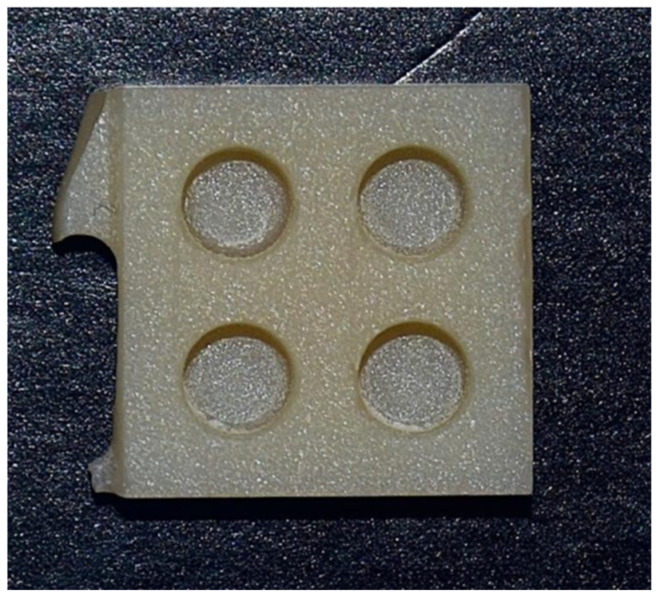
Preparation of the specimen.

An original experimental set was created to perform this study. A total of 15 parallelepiped-shaped specimens (10.5 × 10.5 × 3 mm) with four cavities (∅ 3 mm × depth 2 mm) were designed with an online software (Tinkercad^®^, Autodesk, San Francisco, CA, USA), and the obtained standard tessellation language (STL) file was imported into the InLab^®^ CAM (Dentsply Sirona, Charlotte, NC, USA) software program (Figure 1). Each specimen was milled (Figure 2) with Cylinder Pointed Burs 12S and Step Burs 12S (Dentsply Sirona, Charlotte, NC, USA) in a precision chairside wet milling machine, CEREC^®^ MC XL (Dentsply Sirona, Charlotte, NC, USA). For each specimen, a new set of burs was used, the water tank was pulled out from the milling unit, the filter was replaced, and new distilled water and lubricant (DENTATEC CEREC/inLab; Dentsply Sirona, Charlotte, NC, USA) were filled into the tank. The specimens were consequently subjected to the crystallization process, as indicated by the manufacturer, in a SpeedFire^®^ furnace (Dentsply Sirona, Charlotte, NC, USA).

### 2.2. Surface Treatment

The inner surfaces of each cavity were etched using 5% hydrofluoric acid (HF; IPS Ceramic Etching Gel, Ivoclar, Schaan, Liechtenstein) for 20 s, rinsed with water for 30 s, and then dried with air blowing. Then, silane coupling agent (Silane, Ultradent, South Jordan, UT, USA) was applied for 1 min, followed by air blowing. The universal adhesive system Futurabond U (Voco, GmbH, Cuxhaven, Germany) was applied to the cavities with the aid of a microbrush, using friction movement on the ceramic structure for 20 s. A brief jet of air was then applied over the bonding agent for 5 s before the adhesive system was light cured for 20 s using an LED curing unit (LCU; VALO Corded, Ultradent, UT, USA) with an irradiance of 1200 mW/cm^2^. The cavities were filled with the investigated RBCs (*n* = 12), applying two increments. The last increment was covered with a Mylar strip and pressed using a glass slide with a constant 1 kg pressure to obtain a flat surface. The composite restorations were polymerized for 20 s with the LED held centered over each cavity. After curing, restorations were polished by the same operator using Sof-Lex DiscsTM (3M ESPE, St. Paul, MI, USA). A medium, fine, and extra-fine grit sequence of Sof-Lex was used sequentially with mild hand pressure. A new set of discs was used for every specimen.

### 2.3. Instrumental Evaluation

**Figure 3:**
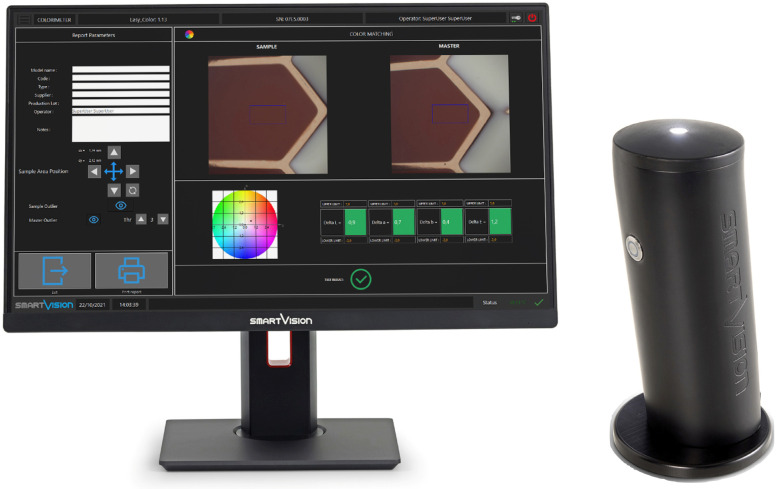
The professional benchtop colorimeter used in this study.

Color parameters were measured, after 72 h of storage in distilled water, using a professional benchtop colorimeter, Easy Color (SmartVision, Udine, Italy) (Figure 3).

The settings were as follows: illuminant: *D*; and temperature: 50 with a neutral grey background (ISO/TR 28642 [30]). White and black calibration was performed as suggested by the manufacturer at the beginning of the test and after each group was completed.

Measurements were performed at two points:

- At the center of resin composite restoration;
- At the center of the CAD/CAM block at an equidistant position to the 4 cavities.

Measurements were repeated by the same operator three times for each composite restoration and specimen, and the average value over the three measurements was calculated. The CIE XYZ tristimulus values were converted to CIE L*a*b* parameters using a dedicated software (SmartVision Easy Color, Udine, Italy): L* refers to luminosity (L* = 0 = black; L* = 100 = white), a* indicates the chroma on the red–green axis (a* ≥ 0 = red and a* ≤ 0 = green), and b* the chroma on the yellow–blue axis (b* ≥ 0 = yellow; b* ≤ 0 =blue). Color differences were expressed in ∆E_00_ units using the CIEDE 2000 color difference according to the following equation [31,32,33]:

(1) $$\Delta E_{00}={\left[{\left(\frac{∆L^{\prime }}{k_{L}S_{L}}\right)}^{2}+{\left(\frac{∆C^{\prime }}{k_{C}S_{C}}\right)}^{2}+{\left(\frac{∆H^{\prime }}{k_{H}S_{H}}\right)}^{2}+R_{T}\left(\frac{∆C^{\prime }}{k_{C}S_{C}}\right)\left(\frac{∆H^{\prime }}{k_{H}S_{H}}\right)\right]}^{\frac{1}{2}}$$

where ΔL′, ΔC′, and ΔH′ are the differences in lightness, chroma, and hue, respectively, for a pair of points. The weighting functions (SL, SC, and SH) adjust the total color difference for variation in the location of the color difference pair in the L′, a′, and b′ coordinates. The parametric factors (k_L_, k_C_, and k_H_) were set to 1. The three ∆E_00_ values obtained for each tested material were averaged to obtain one mean ∆E_00_ value for each RBC. Color adaptability was assessed based on the color differences between the lithium disilicate block and composites across the threshold of 50%:50% perceptibility (PT) and 50:50% acceptability (AT) according to ISO/TR 28642:2016. The values ∆E_00_ = 0.8 and ∆E_00_ = 1.8 were reported as the 50:50% perceptibility threshold (PT) and 50:50% acceptability threshold (AT) [34].

### 2.4. Statistical Analysis

The normal distribution of data was verified using the Shapiro–Wilk test, and the homogeneity of variance was assessed using the Brown–Forsythe test. Mean values were compared to determine which of the five tested materials was the closest match using a one-way analysis of variance (ANOVA) test. All analyses were performed with the statistical platform STATA (STATA, v12.0, StataCorp, College Station, TX, USA). For the current study, the level of significance (α) is 5%. Hence, *p* ≤ 0.05 was considered significant.

## 3. Results

**Figure 4:**
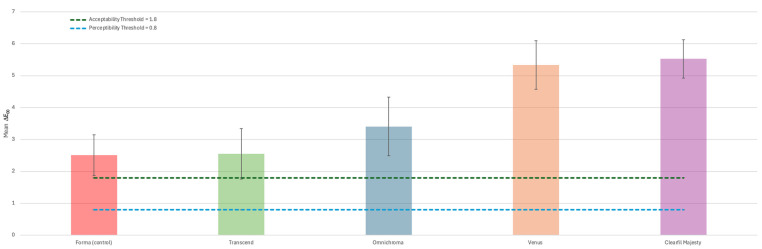
Column chart showing the mean ΔE_00_ for the tested materials. The black vertical lines represent the standard deviation for each group. The green dashed line represents the acceptability threshold (ΔE_00_ = 1.8) and the blue dashed line represents the perceptibility threshold (ΔE_00_ = 0.8). Shorter columns show better integration.

One-way analysis of variance (ANOVA) showed significant differences (*p* < 0.05) in the ability of materials to blend with the substrate. Table 2 and Figure 4 both present the mean color differences (ΔE_00_) and standard deviations (SDs) between the baseline lithium disilicate block and restored cavities for each group. All restorations with the tested materials presented color difference levels above the acceptable threshold (AT = 1.8). Among the study groups, CM showed the highest ΔE_00_ (5.53 ± 0.60), while F, a multi-shade RBC, showed the lowest (2.51 ± 0.79). For all other groups, ΔE_00_ ranged from 2.55 ± 0.64 to 5.34 ± 0.76, with significant differences (*p* < 0.05). The results showed there was a statistically significant difference between the control group G1 and the tested groups G3, G4, and G5 (*p* < 0.05). Pairwise comparisons showed significant differences (*p* < 0.05) between G3 and G4, between G3 and G5, between G2 and G4, between G2 and G5, and between G4 and G5. No significant difference was found between the control group G1 and G2 and between G2 and G3 (*p* > 0.05).

A clear trend emerged: materials with defined shades or with fillers chemically similar to the lithium disilicate substrate (e.g., Transcend) tended to show better color matching. All groups, however, exceeded the clinical acceptability threshold, underscoring limitations in the universal shade concept under these testing conditions. No anomalies or outliers were observed, and data showed normal distribution and homoscedasticity.

## 4. Discussion

To reduce operative time and simplify clinical procedures, which are often too technique-sensitive, the industry has invested in developing single-shade universal composites [8]. Manufacturers claim that these new composites can mimic the color of the surrounding tooth regardless of its shade. The objective of this study was to instrumentally evaluate the chameleon effect of four commercially available universal shade composites compared to a multi-shade resin-based composite (RBC) used for the repair of lithium disilicate prosthetic elements. The null hypothesis was rejected, as statistically significant differences were observed between the control and the tested groups. The results showed that the ∆E_00_ values for all groups exceeded the perceptibility (PT) and acceptability (AT) thresholds, indicating that the shade mismatches were not only perceptible but also clinically unacceptable. Among all tested materials, Forma (F) demonstrated significantly better color adjustment (∆E_00_ = 2.51) than the universal composites, except for Transcend (T) (∆E_00_ = 2.55). The average ∆E_00_ value of the control group was closer to the acceptability threshold than those of the universal shade composites. De Abreu et al. [35] and Korkut et al. [36] experimented on the color adjustment potential of composites in class III cavities and class I cavities, respectively. In both these in-vitro studies, RBCs with a defined shade (e.g., A1, A2, A3, etc.) showed better color matching. The results reported in our study are consistent with their findings. Forma’s superior blending may be attributed to two main factors: shade selection and translucency. Paravina et al. [7] determined that the BE increases when there is less color difference between the RBCs and the surrounding structure. Amid the materials tested, the control group was the only one with a defined shade (A3) that matched the substrate. The use of specific color pigments may have enhanced the blending capability of the control group. Villarroel et al. [37] have shown that translucency plays a key role in the perceived color of a restoration. Various factors [38] can influence translucency and consequently, the blending behavior of resin composites. These include material thickness, shade, background environment, resin matrix composition, filler content and size [39], and refractive index (RI) [40]. The RI of a material affects light propagation and thus the perceived color of the restoration. A close match in RI between the resin and surrounding tooth structure results in minimal light scattering [40,41], leading to higher translucency and improved blending. Conversely, a mismatch in RI can cause shadow-like effects at the restoration margins due to destructive light interference between materials with different RI values [37,42]. Therefore, to achieve a natural appearance, dental composites should have an RI as close as possible to that of natural enamel [37,43], which has a reported mean RI of 1.631 [44]. The average RI of lithium disilicate is 1.55 in the visible light spectrum [45], while that of Bis-GMA is 1.54 [46]. This close match between the refractive indices of lithium disilicate and Forma may partly explain the superior color matching observed with Forma. However, CM also contains Bis-GMA, yet it exhibited the lowest chameleon effect. The RI of a resin composite is influenced by both the type of resin matrix and the inorganic fillers added [46]. Since F is the only material in this study that includes the specialized filler ytterbium trifluoride—whose RI is very close to that of resin monomers (1.49–1.56) [47]—it is likely that the presence of this filler contributes significantly to its enhanced translucency. Paravina et al. [7] reported that the BE increases with greater translucency, supporting the hypothesis that the addition of ytterbium trifluoride may explain Forma’s superior chameleon effect in this study. Nevertheless, RI is not the only factor to consider in achieving esthetic restorations. Proper thickness is equally important. A linear decrease in RI with increasing specimen thickness was observed by Son et al. [48]. Further research varying the thickness of single-shade specimens could help quantify the impact of this variable on RI. Among the universal shades, Transcend (T) showed the lowest ∆E_00_ value, while CM showed the highest. The high color match of T may be attributed to its inclusion of five different types of silicate fillers—chemically similar to those in lithium disilicate—potentially allowing a more mimetic repair. Conversely, CM’s poorer color match could be due to its larger filler particles (mean size of 1.5 µm), which tend to scatter more light and reduce transmittance [49,50]. OC, in comparison, showed a lower blending effect than T, though without statistically significant differences. OC does not contain Bis-GMA in its resin matrix and incorporates uniformly sized 260 nm spherical silicon/zirconium dioxide fillers. It also features a structural color-based shading system, meaning it achieves color through the physical structure of its fillers rather than through added pigments. This may have affected its color blending performance compared to the other materials evaluated.

Our findings contrast with those of Pereira Sanchez et al. [8], who reported a greater color-matching ability for OC. However, their study evaluated OC in posterior occlusal restorations and used the CIELab formula, which may account for the discrepancy. Recent studies have concluded that the CIEDE2000 formula more accurately reflects human visual perception than the traditional CIELab color difference metric [51,52,53,54]. This difference may account for the discrepancies between studies. Conversely, our findings align with those of Iyer et al. [55]. They found that OC exhibited inferior color-matching ability compared to a resin composite with a defined shade. They also observed that OC visually matched better with lighter shades. Islam et al. reported [56] that OC blended more effectively with shades such as B1, A2, and B2 of CharmFil Plus (CP) and Filtek Universal Restorative (3M), but showed greater contrast with darker shades like A3 and A3.5. Since shade A3 was used in our study, this could partially explain OC’s lower CAP. Akgul et al. [57] demonstrated that increasing the cavity size enhanced OC’s blending effect, likely due to changes in the optical behavior of surrounding tooth structures. They concluded that 3.0 mm restorations achieved better color matching than 2.0 mm ones. In our experiment, standardized cavities with a depth of 2 mm and diameter of 3 mm were selected to ensure complete curing of the RBCs. Further studies varying cavity depth could help determine whether restoration size influences the blending performance of universal shade composites. Additionally, the study by Korkut et al. [36] reported improved CAP of OC and Essentia Universal after two weeks compared to the baseline measurements. These findings underscore the need for long-term evaluations to assess the durability of the chameleon effect. Surface treatment may also affect the optical blending behavior of universal shades. In the study by Karabulut et al. [58], a higher color difference for OC in Sylc-treated Empress blocks was observed, while Essentia Universal exhibited greater color shifts in Sylc-treated Cerasmart270 CAD blocks. The authors attributed these differences to the adhesion of Sylc particles, which may interact differently with various chromatic technologies. Therefore, further investigation into how surface treatments influence color performance is warranted. Similarly, the type of adhesive system can impact the final shade of the restoration. According to Schneider et al. [59], adhesives may alter color parameters, particularly along the a and b axes, largely due to their chemical composition. Oxidative reactions involving Camphorquinone and amine components—such as those found in Futurabond U—can lead to darkening and increased yellowing over time. Beyond differences in filler content and translucency, deeper material science factors may explain the superior blending of certain RBCs. One critical aspect is the homogeneity of the resin–filler interface: a well-integrated interface minimizes internal light scattering and promotes more uniform light transmission. In composites with nano- or submicron fillers, such as Transcend, light scattering may occur in the Rayleigh regime, where intensity is inversely proportional to the fourth power of the wavelength [60]. This selective wavelength scattering can enhance visual blending with adjacent structures. Moreover, the stability and distribution of filler particles within the resin matrix influence not only the mechanical properties but also the optical phase continuity—governing how light transitions between resin and filler phases [60,61]. Composites with high optical phase continuity, minimal refractive index mismatches, and optimized filler–resin coupling are more likely to deliver superior optical blending [62]. These findings suggest that the chameleon effect is influenced by both translucency and the nanoscale architecture of the composite.

When performing repairs of lithium disilicate restorations, clinicians should be aware that not all universal shade composites provide comparable blending performance. Materials with defined shades—such as Forma (A3)—demonstrated superior optical integration due to better shade matching and refractive index compatibility. Among universal composites, those containing finer filler particles and chemically similar filler compositions to lithium disilicate, such as Transcend, may provide better esthetic results. Clinicians are advised to consider multiple factors when selecting a repair composite, including the shade of the ceramic substrate, the translucency of the composite, and the material’s refractive properties [63]. Whenever possible, visual evaluation and shade matching before initiating the repair procedure are recommended to optimize esthetic outcomes.

The clinical implications of the current study are as follows: using defined-shade composites in shade-specific repairs improves the esthetic integration of the restoration; although universal composites offer workflow simplicity, they may not meet patients’ esthetic expectations in visible areas; and visual shade selection and pre-curing evaluation are recommended to confirm the suitability of the selected composite.

This study evaluated the chameleon effect of universal shade composites in repairing CAD/CAM lithium disilicate restorations; however, several limitations should be acknowledged. The in vitro nature of the research does not fully replicate clinical conditions, where factors such as saliva, observer variability, absence of aging and thermal cycling, temperature variations, and occlusal forces may affect the blending ability and long-term stability of the restorations. Moreover, only one shade of lithium disilicate (HT A3) was tested, whereas the color adaptation of universal composites could vary depending on the background shade and translucency. Another limitation is the short-term assessment, as color measurements were performed immediately after restoration placement, without considering aging effects such as wear, staining, and potential discoloration over time. To address the limitations inherent in this in vitro, short-term study, future research should incorporate clinically relevant aging simulations and visual assessments. Specifically, thermocycling and artificial staining protocols could be applied to evaluate the durability of the chameleon effect under intraoral-like conditions. Additionally, long-term monitoring of color stability over periods of 1–6 months would help quantify time-dependent changes in blending behavior. Including subjective visual assessments by trained evaluators—alongside instrumental colorimetry—would offer a more comprehensive evaluation of esthetic performance. These strategies would help bridge the gap between laboratory findings and real-world clinical applicability. Further studies are warranted to explore the impact of cavity depth and size on the blending effect, as well as investigate different surface treatment protocols and adhesive systems that may influence optical behavior. Additionally, color assessment in this study was instrumentally performed, without accounting for human visual perception, which plays a key role in clinical esthetics. Incorporating both objective and subjective evaluations would provide a more comprehensive understanding of color matching.

Long-term clinical trials are essential to validate the findings from in vitro experiments and assess the durability and overall performance of universal shade composites in real-world conditions.

## 5. Conclusions

Within the limitations of the present study, it can be concluded that among universal composites, Transcend approached the performance of the multi-shade material but still exceeded the acceptability threshold under these test conditions. These outcomes indicate that universal shade composites do not uniformly achieve reliable blending with ceramic substrates. Clinicians should consider material composition and optical compatibility when esthetics are critical.

## Figures and Tables

**Table 1:** Manufacturers’ information about the RBCs.

Materials	Manufacturer	Shade	Monomers	Filler Size/Content	Filler [*w*/*v*%]
Omnichroma^®^ (OC) G3	Tokuyama Dental Corporation, Tokyo, Japan	Universal	UDMA, TEGDMA	SiO_2_, ZrO_2_, CF	79/68
Transcend^®^ (T) G2	Ultradent Products Inc., South Jordan, UT, USA	UB	5 different resin monomers described as functional methacrylates	Five different types of silicate fillers, ranging in size from 5 nm to 3 μm	77.5/na
Venus Pearl^®^ (V) G4	Heraeaus Kulzer, Hanau, Germany	One	UDMA, TCD-DI-HEA, TEGDMA	Ba-Al-B-F-Si, PPF, SiO_2_	75/59
Clearfil Majesty Universal^®^ (CM) G5	Kuraray Noritake Dental, Osaka, Japan	Universal	Bis-GMA, Hydrophobic aliphatic dimethacrylate, Hydrophobic aromatic dimethacrylate, di-camphorquinone	Silinated barium glass filler	78/66
Forma^®^ (F) G1	Ultradent Products Inc, SP, Brazil	A3B	Bis-GMA, Bis-EMA, TEGDMA, BHT, PEGDMA, UDMA, ytterbium trifluoride	Silane-treated ceramic, silane-treated silica, silane-treated silica–zirconium oxide and barium glass	67/na
CEREC Tessera^®^ HT	Dentsply Sirona, Charlotte, NC, USA	A3	90% lithium disilicate (Li_2_Si_2_O_5_), 5% virgilite (Li_0.5_Al_0.5_Si_2.5_O_6_) 5% Li_3_PO_4_		

**Table 2:** Mean of colors difference values (ΔE_00_) and SDs for the five composite materials tested in the study. The same letters (A to D) represent no statistically significant differences (*p* > 0.05).

Groups	ΔE_00_ Mean and Standard Deviation
G1 (F; control)	2.51 ± 0.79 ^A^
G2 (T)	2.55 ± 0.64 ^AB^
G3 (OC)	3.41 ± 0.92 ^B^
G4 (V)	5.34 ± 0.76 ^C^
G5 (CM)	5.53 ± 0.60 ^Ds^

## Data Availability

The raw data supporting the conclusions of this article will be made available by the authors on request.

## Abbreviations

The following abbreviations are used in this manuscript:

RBCs	Resin-based composites
OC	Omnichroma
CM	Clearfil Majesty Universal
V	Venus Pearl
T	Transcend
F	Forma
BE	Blending effect
CAP	Color adjustment potential
HF	Hydrofluoric acid
LCU	LED curing unit
AT	Acceptability threshold
PT	Perceptibility threshold
SDs	Standard deviations
RI	Refractive index
